# Cross-species comparison of CAR-mediated procarcinogenic key events in a 3D liver microtissue model

**DOI:** 10.1016/j.toxrep.2019.09.010

**Published:** 2019-09-24

**Authors:** Simon Plummer, Bobby Beaumont, Stephanie Wallace, Graeme Ball, Jayne Wright, Liz McInnes, Richard Currie, Rich Peffer, David Cowie

**Affiliations:** a MicroMatrices Associates Ltd, Dundee, UK; b Dundee University Imaging Facility, Dundee, UK; c Jayne Wright ltd, Putley, UK; d Syngenta ltd, Bracknell, UK; e Syngenta Inc, Greensboro, USA

**Keywords:** 3D liver microtissues, Tissue microarray, Constitutive androstane receptor, Key events, Carcinogenesis, Quantitative histopathology, Cross-species risk assessment, Transcriptomics, Proteomics, Hepatocyte, Mode of action, Proliferation, Enzyme induction, Pathways, MicroTMA, Species difference

## Abstract

Characterisation of the mode of action (MOA) of constitutive androstane receptor (CAR)-mediated rodent liver tumours involves measurement 5 key events including activation of the CAR receptor, altered gene expression, hepatocellular proliferation, clonal expansion and increased hepatocellular adenomas/carcinomas. To test whether or not liver 3D microtissues (LiMTs) recapitulate CAR- mediated procarcinogenic key events in response to the prototypical CAR activator phenobarbital (PB) we performed hepatocyte proliferation (LI%) analysis in rat and human LiMTs using a microTMA technology in conjunction with integrated transcriptomics (microarray) and proteomics analysis. The rationale for this approach was that LiMTs containing parenchymal and non-parenchymal cells (NPCs) are more physiologically representative of liver and thus would generate data more relevant to the in vivo situation. Rat and human LiMTs were treated with PB over a range of concentrations (500 uM - 2000 uM) and times (24 h - 96 h) in a dose-response/time-course analysis. There was a dose-dependent induction of LI% in rat LiMTs, however there was little or no effect of PB on LI% in human LiMTs. ATP levels in the rat and human LiMTs were similar to control in all of the PB treatments. There was also a dose- and time-dependent PB-mediated RNA induction of CAR regulated genes CYP2B6/Cyp2b2, CYP3A7/Cyp3a9 and UGT1A6/Ugt1a6 in human and rat LiMTs, respectively. These CAR regulated genes were also upregulated at the protein level. Ingenuity pathways analysis (IPA) indicated that there was a significant (Z score >2.0;-log p value >) activation of CAR by PB in both human and rat LiMTs. These results indicate that human and rat LiMTs showed the expected responses at the level of PB-induced hepatocyte proliferation and enzyme induction with rat LiMTs showing significant dose-dependent effects while human LiMTs showed no proliferation response but did show dose-dependent enzyme induction at the RNA and protein levels. In conclusion LiMTs serve as a model to provide mechanistic data for 3 of the 5 key events considered necessary to establish a CAR-mediated MOA for liver tumourigenesis and thus can potentially reduce the use of animals when compiling mechanistic data packages.

## Highlights

• The first demonstration of phenobarbital-induced hepatocyte proliferation in 3D liver microtissue models.
• Integration of quantitative histopathology data with genomics (transcriptomics/proteomics).
• In vitro cross-species risk assessment.
• CAR-mediated mode of action.
• Quantitative histopathology of 3D microtissues.

## 1 Introduction

Liver tumours are a common occurrence in rodent 2 year bioassay tests of drugs and pesticides. In some cases the process of carcinogenesis involves activation of nuclear hormone receptors via mechanisms that are not considered relevant to humans [1]. One such mechanism involves activation of the constitutive androstane receptor (CAR) [2]. Recent methods for testing this mode of action involving modified Bradford Hill considerations of causality have established five key events in the process of CAR - mediated liver carcinogenesis: 1. Activation of CAR nuclear receptor; 2. Altered gene expression secondary to CAR activation; 3. Increased hepatocellular proliferation; 4. Increased clonal expansion leading to increased altered foci and 5. Increased hepatocellular adenomas, carcinomas [3,4]. A stepwise scheme for this purpose involving establishment of key events and associated events in the process has been outlined in the International Programme on Chemical Safety (IPSC) framework. The approach also facilitates assessment of human relevance of the mode of action by incorporating a comparison of key events in rodent and human in vitro models.

Current in vitro methods for rodent versus human comparisons of CAR-mediated liver carcinogenesis key events have utilised 2D cultures of primary hepatocytes [4]. Whilst 2D cultures of primary hepatocytes have in many cases recapitulated CAR ‘key event’ responses such as hepatocyte proliferation and enzyme induction [5,6] these cultures rapidly lose CAR responsiveness after a few days in culture [7,8]. As certain key event and associated event responses to CAR activation in vivo by prototypical CAR activators, e.g. phenobarbital (PB), such as CAR activation and enzyme induction occur over long term (weeks) exposures [9,10] the use of 2D cultures as models for the in vivo situation is suboptimal. 3D liver microtissue models containing primary hepatocytes and non-parenchymal cells, unlike 2D primary hepatocytes, retain 'liver like' gene expression profiles and also sustain responsiveness to enzyme inducers such as phenobarbital (PB) for several weeks in culture [11]. Hence these models offer the potential to provide an in vitro system that more closely represents in vivo liver responses. To test this hypothesis we utilised novel high throughput histology, transcriptomics and proteomics approaches to investigate three ‘key event’ responses in CAR mediated carcinogenesis, namely CAR activation, enzyme induction and hepatocyte proliferation in a 3D liver microtissue model.

## 2 Materials and methods

### 2.1 Liver microtissue (LiMT) treatments

**Fig. 1:**
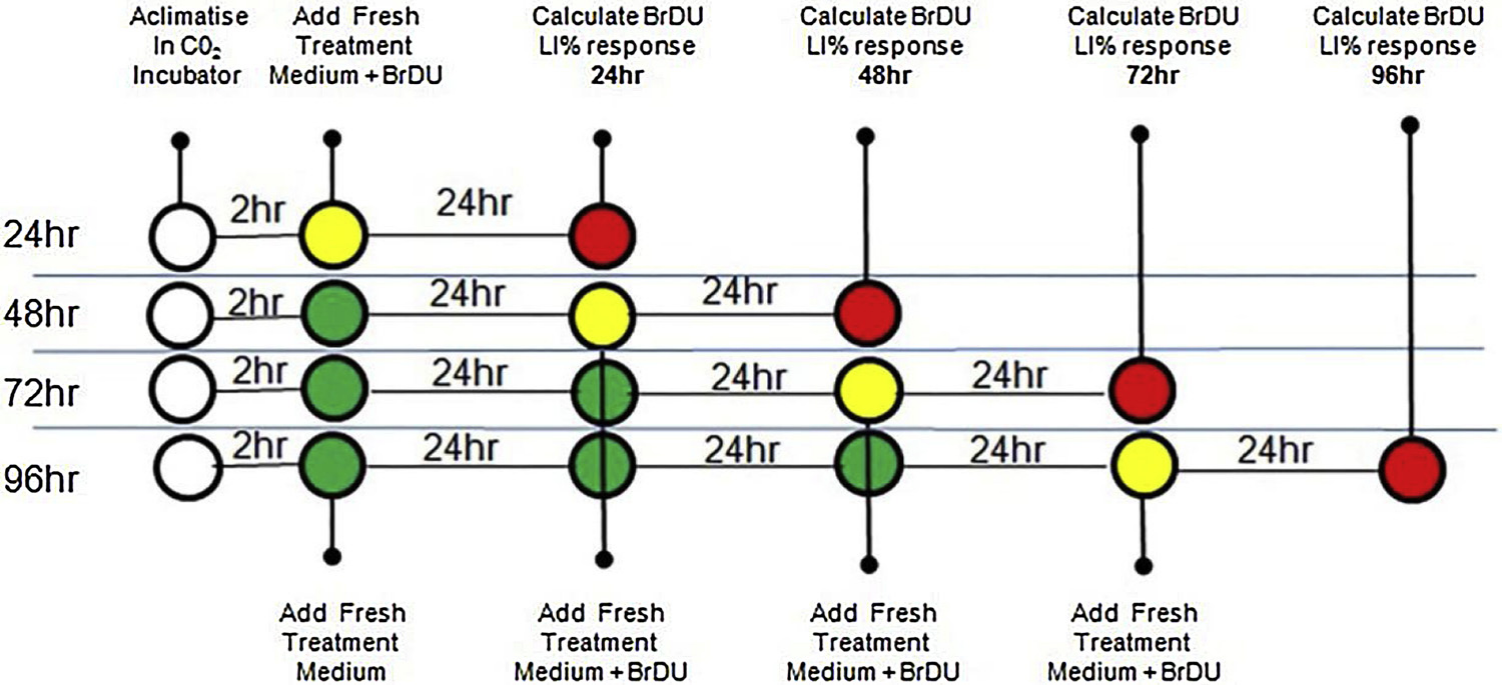
Schematic showing the treatment schedule of rat and human LiMT plates in both the enabling and main studies. Green dots show addition of treatment media without BrDU. Yellow dots show addition of the treatment media plus BrDU which was added for last 24 hs of the treatment interval. Red dots show harvesting the tissues at the end of the 24, 48, 72 and 96 h intervals.

InSphero LiMT rat (primary hepatocytes, co-cultured with NPCs (InSphero #MT-02-00104) and human LiMT (multi-donor primary hepatocytes, co-cultured with NPCs (InSphero #MT-02-302-04) were treated with a stock solution of 200 mM PB in fresh 3D Insight rat liver maintenance medium (InSphero #CS-07-002-01) and 3D Insight human liver maintenance medium-AF (InSPhero #CS-07001a-01), respectively, to give final concentrations of 500 uM, 750 uM, 1 mM and 2 mM; an HGF (Sigma #H9661-5UG) positive control was included at a concentration of 50 ng/ml (rat) and 100 ng/ml (human). The rat and human LiMTs were exposed, one spheroid per well, in a 96 well gravity trap™ plate, to PB dissolved in rat or human liver maintenance medium, respectively, for four different time periods: 24 h, 48 h, 72 h and 96 h. Experiments were performed at least twice on different batches of spheroids. 24 h prior to fixing a media change was performed using media containing BrDU (10 uM) (Sigma #B5002-100 mg), Fig. 1. After incubation of the spheroids for the appropriate periods of time, LiMTs were fixed in 4% paraformaldehyde [Pierce 16% paraformaldehyde (Thermo Fisher Scientific #28908)], diluted in DPBS (ThermoFisher #14190-094) for 30 min at room temperature after which they were rinsed in DPBS.

### 2.2 Spheroid tissue microarray (microTMA) construction

To facilitate high-throughput histology analysis of LiMTs, paraformaldehyde (4%) fixed spheroids from each drug treatment were systematically organised in a microTMA using a previously published method (MicroMatrices international patent application No. PCT/GB2016/053907 publication number WO 2017/174955; Plummer et al 2019). Briefly, fixed spheroids were loaded into the wells of a 2% agarose mold containing 96 wells and sealed using molten 0.7% agarose. We embedded 16 spheroids per treatment in the microTMA mold. The agarose mold containing spheroids was dehydrated for a minimum of 12 h in 70% ethanol and then the microTMA mold was processed to paraffin wax in a tissue processor (Thermo Citadel 1000). Following wax embedding the microTMA block was sectioned (6 uM sections) using a microtome (Reichert Jung) onto glass microscope slides.

### 2.3 Immunostaining

MicroTMA slides were dewaxed in Histoclear and then heat induced epitope retrieval (HIER) was performed using citrate buffer solution pH6 (Vector labs). Parallel sections were either haematoxylin and eosin stained or immunostained. Anti-BrDU dual immunofluorescence (IF) staining was performed as follows: microTMA slides were dewaxed (Histoclear), rehydrated through graded ethanols (100%-70%) and subjected to HIER in pH 6.0 citrate buffer for 20 min at 121 °C in an antigen retriever (Prestige Medical). After HIER, the slides were washed in distilled water, and mounted in PBS on a Shandon disposable immunostaining chamber according to the manufacturer’s instructions. Blocking buffer (1% BSA (Sigma A7906-100 G), 0.2% Triton X 100 (VWR #3063324), 3% normal goat serum (Sigma G9023-10 ml) in PBS) was added to the chamber and the slides incubated for 2 h at RT. Anti-BrDU (Abcam #ab152095) primary antibody at a 1:200 dilution (PBS/0.1% BSA, 0.5% normal goat serum, 0.2% Triton), which included a primary antibody negative control (wash buffer alone), was then added to the chambers and the slides incubated at RT for 1 h. Slides were washed in wash buffer (0.1% BSA, 0.2% Triton, 0.5% normal goat serum in PBS). The secondary antibody goat anti-rabbit Alexa fluor 568 (Life Technologies # A11011) diluted at 1:500 in wash buffer, was added to the chamber and the slides incubated for 2 h at RT. The slides were then washed in wash buffer and Sytox green nuclear stain (Life Technologies #S7020) diluted at 1:1000 in wash buffer was added to the chamber and the slides were incubated for 5 min. The slides were washed in PBS and mounted using antifade mountant (Vectashield, Vector Laboratories # H-1000). The IF-stained slides were imaged using a Zeiss 710 LSM confocal microscope.

### 2.4 Image analysis

ImageJ (FIJI) software was used for image analysis. Cell counts were based on detection of nuclear staining by the nuclear fluorescent dye Sytox green. Each image was thresholded in the Sytox green channel and quantitation of selected nuclear objects (Sytox green stained) was performed based on size (excluding objects too small or large for hepatocyte nuclei) using filters designed to exclude non-parenchymal cells. Thresholded and selected nuclei were counted using a script in Image *J* (FIJI) software. The BrdU labelling index (LI%) for rat or human hepatocytes was calculated using the following equation:

LI (%) = 100 × (Number of Labelled Nuclei - BrdU+)/(Total number nuclei in spheroid section– Sytox green)%

### 2.5 RNA extraction and transplex labelling

2-4 whole fixed spheroids for each treatment were lifted from the 96 well LiMT plates. RNA from these samples was extracted using the Qiagen *mi*RNeasy FFPE Kit according to the Qiagen RNeasy FFPE Handbook 09/2011. The RNA yield was quantified using a Nanodrop spectrophotometer and extracted RNA was treated with DNase to remove any trace of genomic DNA. 50 ng of RNA was used to generate amplified cDNA with the Sigma TransPlex Whole Transcriptome Amplification system.The cDNA was purified then quantified via Nanodrop spectroscopy. 1800 ng of cDNA generated in the transplex reaction was labelled using the Agilent SureTag DNA labelling Kit and the specific activity (pmol Cy3/ug cDNA) and concentration (ug/ml) of labelled cDNA were quantified by NanoDrop spectroscopy. A specific activity of 15–50 pmol Cy3/ug DNA was considered sufficient for hybridisation on Agilent arrays.

### 2.6 Microarray hybridisation and scanning

1800 ng of purified Cy3-labeled cDNA samples were combined with Agilent-CGH block and Agilent 2x Hi-RPM Hybridisation Buffer (components of the Agilent Oligo aCGH/ChiP-on-chip Hybridisation kit). Hybridisation to Agilent Whole Genome Expression microarrays was performed at 20 RPM at a temperature of 65 °C for 17 h. Washing steps were performed according to the Agilent Oligonucleotide Array-Based CGH for Genomic DNA Analysis Protocol. The Agilent Scanner was equipped with extended dynamic range (XDR) software and Agilent Feature Extraction Software was used for the data extraction from raw microarray image files to generate raw microarray data. Raw microarray data was normalised using R 2.15.2 for Windows with a quantile algorithm using the following packages:“BiocLite.R”, “limma” version 3.14.0, “Agi4 × 44PreProcess” version 1.22.0. All packages used default analysis parameters.

### 2.7 CAR signature pathways analysis of differentially expressed gene (DEG) lists

Following statistical analysis, Ingenuity Pathways Analysis (IPA) was used to identify pathways regulated by the induction of CAR (constitutive androstane receptor). A focussed IPA overrepresentation analysis against a CAR signature gene list derived by Oshida and co-workers (Oshida et al., 2015) was performed. The Oshida CAR signature gene list was imported into IPA and a Tox Analysis was performed against both rat and human DEG data in order to test the effect of treatments on the overrepresentation (Fisher’s Exact Test -log p values) of this pathway in the DEG lists. As IPA overrepresentation (Fisher's exact test) analysis does not take into account the polarity of gene expression changes in the CAR regulated genes in the DEG lists, the polarity of gene expression changes was assessed by reviewing exported Excel data tables for the CAR signature pathway to determine whether or not CAR-regulated genes were induced. The -Log p values in IPA also do not distinguish activation or repression of a pathway. We utilised Z score values in an upstream regulator analysis to assess the significance of activation or repression of the CAR receptor (NR1I3).

### 2.8 Proteomic analysis

Control and PB treated rat LiMTs and human LiMTs (23 spheroids/sample) were extracted and placed into 8 Protein Lo-bind tubes. Rat and Human LiMTs were analysed at the 72 h and 96 h time points, respectively, based on an assessment of the kinetics of PB induction of certain CAR regulated ‘signature’ genes at the RNA level where it was found that there was maximal induction of Cyp2B2 and CYP2B6 at 72 h and 96 h for rat and human LIMTs, respectively (see Results section- enzyme induction). In rat LiMTs, 8 samples (30 spheroids/sample) were generated, of which 4 were controls and 4 were PB treated, and the analysis was performed for control and PB 750 u M treated samples at the 72 h time point.

Protein was extracted from fixed samples using the Qproteome FFPE Tissue kit (QIAGEN) according to the manufacturer's instructions and protein concentration was measured using the EZQ method (EZQ® Protein Quantitation Kit, Invitrogen). Protein was then precipitated by adding methanol and chloroform, followed by overnight Trypsin digestion. Peptides were TMT labelled (TMT 10plex Reagent Kit, Thermo Scientific) and the labelled samples were combined and purified. 4 fractions were produced from the combined sample via offline HPLC on an XBridge BEH C18 Column. These fractions were evaporated to dryness and resuspended in 1% formic acid prior to injection into the Lumos Fusion Orbitrap LC MS/MS followed by analysis to raw data.

### 2.9 Statistical analysis of transcriptomics and proteomics data

Fold change values in the transcriptomics analysis were calculated in R software using a linear model for microarray data analysis (LIMMA), and Benjamini Hochberg multiple test correction was applied to p values derived from the *t*-test analysis to derive q values [false discovery rate (FDR) adjusted p values] for these alterations.This process was used to identify a list of differentially expressed genes (DEG list), relative to control, for each of the treatments using a cut off point for the q value derived from the LIMMA *t*-test set at p < 0.05. Proteomics data was analysed in Proteome Discoverer 2.1 and microsoft Excel softwares. Raw intensity values for each peptide were normalised by taking the channel (isobaric tag) in the 8 plex analysis with the highest total peptide abundance value and then correcting all the other channels by a constant factor so that all channels had the same total abundance value. These normalised values were then scaled for every peptide so that the average of all channels had an intensity of 100 intensity units. A fold change and p value was calculated from 4 replicate data points for each treatment using Student *t*-test in Excel.

### 2.10 Integration of rat and human proteomic and transcriptomic data

Processed data from the transcriptomic experiments was uploaded to IPA simultaneously with the proteomic data produced from Proteome Discoverer analysis. Integration of the transcriptomic and proteomic data was performed subsequent to normalisation and statistical analysis using an R script that combined the data using the Genbank/Uniprot abbreviated gene names. Rat and human integrated datasets were uploaded to IPA for bioinformatics analysis.

## 3 Results

### 3.1 Hepatocyte proliferation

**Fig. 2:**
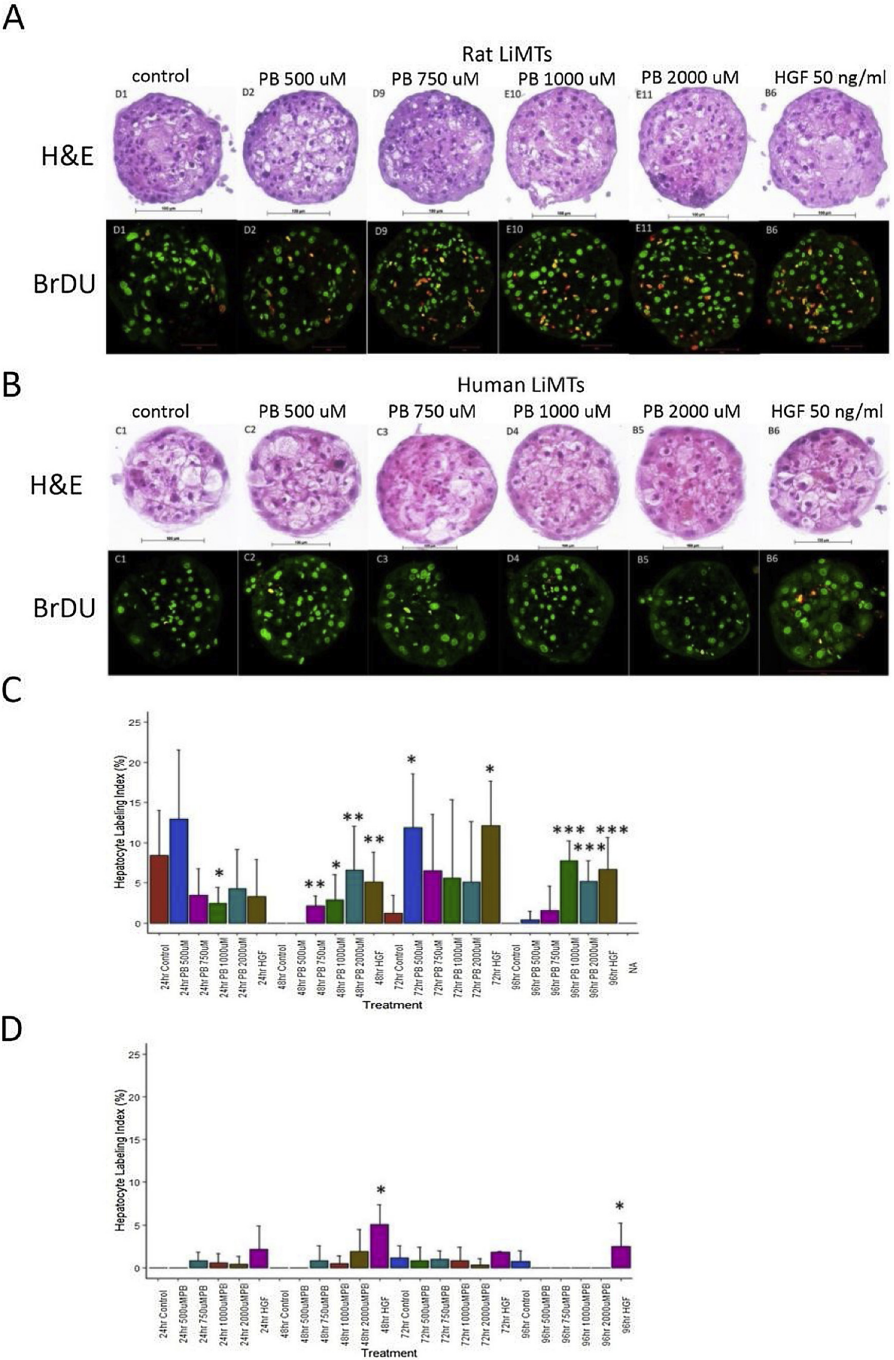
Effect of PB treatments on hepatocyte proliferation (LI%) in rat and human LiMTs. (A) rat and (B) human LiMTs showing representative images of H&E stained (top panels) or anti-BrDU immunofluorescence (IF) stained (bottom panels) LiMTs from parallel microTMA sections. (C) and (D) are histograms of hepatocyte proliferation (LI%) data derived from image analysis of BrDU IF stained rat and human LiMT sections, respectively. Results are mean ± SD, n = 4-8. *, **, *** significantly different from control by Student's t test, p < 0.05, p < 0.001, p < 0.0001, respectively. The data ware representative of at least two separate experiments performed on different batches of spheroids.

There was a dose- and time-dependent induction of hepatocyte proliferation (LI%) in rat LiMTs at the 48 h, 72 h and 96 h time points which peaked at the 96 h time point, Fig. 2C. At the 24 h time point there was a high basal level of proliferation in the control rat LiMT samples which may be attributed to a stress response caused by assembly/shipment of the spheroids. By contrast there was no significant induction of LI% by PB at any of the doses or time points in human LiMTs, Fig. 2D. Hepatocyte growth factor (HGF) treatments caused a significant induction of LI% in both rat and human LiMTs, Fig. 2C and D, respectively. The levels of ATP in rat and human LiMTs following PB treatments were not significantly different from control indicating that the PB treatment did not cause cytotoxicity, Table 1. Histopathological examination of H&E sections of the spheroids following the treatments also showed no signs of central necrosis, Fig. 2A and B. There was some vacuolation in the hepatocytes of both rat and human LiMTs but the finding is not considered significant in view of the functional response to PB evidenced by the induction by PB of CAR regulated genes at the RNA and protein level (see below).

**Table 1:** ATP levels in the rat and human LiMTs after PB (500 u M, 750 u M, 1000 u M and 2000 u M) treatments. Results are means ± SD, n = 2-4. N/D = not determined.

Treatment	Rat LiMTs	Human LiMTs
	ATP pmoles/LMT	ATP pmoles/LMT
control	11.8 ± 1.1	15.2 ± 5.1
PB 500 u M	17.4 ± 0.3	N/D
PB 750 u M	18.6 ± 2.0	15.0 ± 4.8
PB1000 u M	19.2 ± 2.0	13.8 ± 4.8
PB 2000 u M	20.1 ± 2.4	16.0 ± 2.8

### 3.2 Enzyme induction

**Fig. 3:**
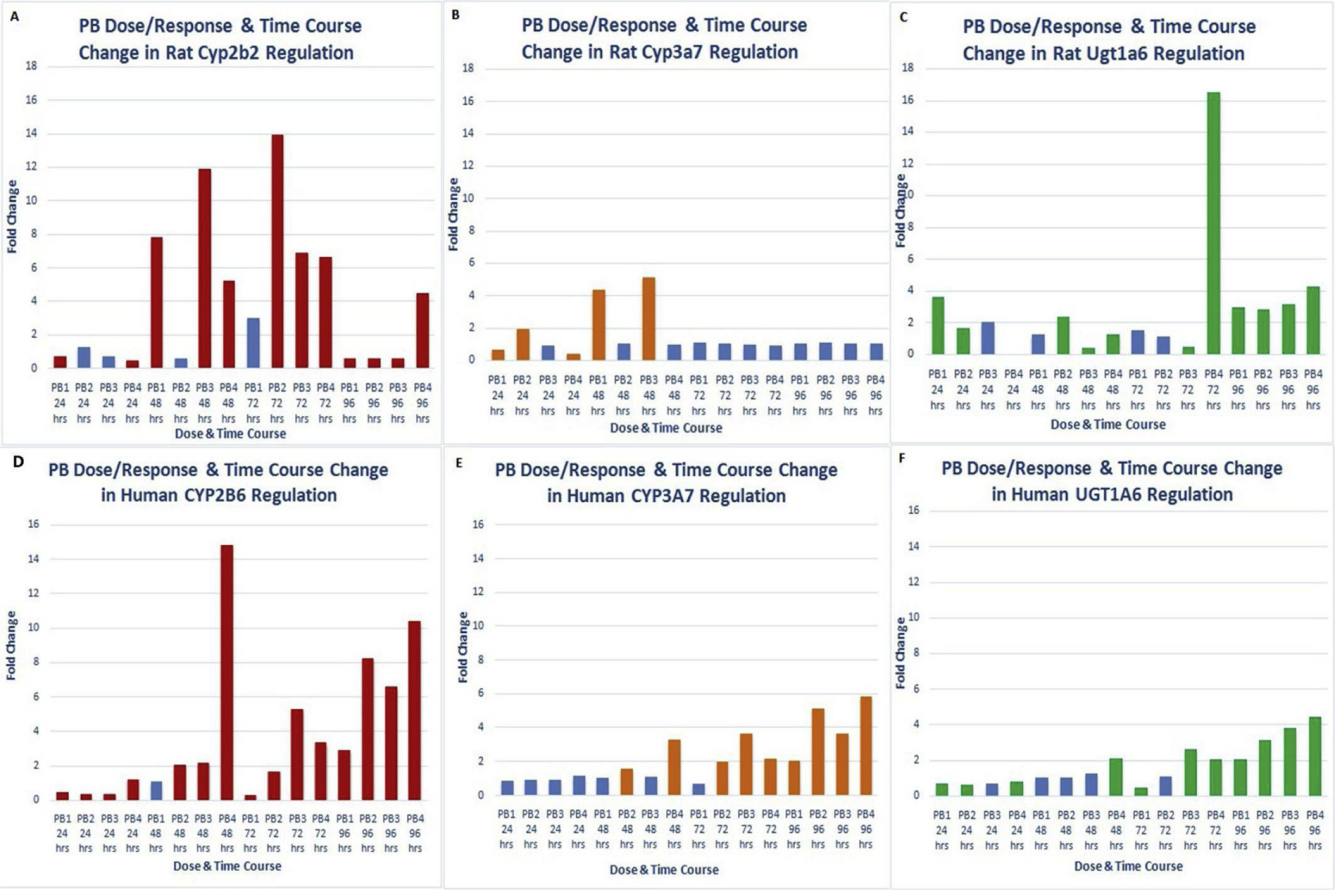
Effects of PB treatment over time on the induction (fold change) of CAR-regulated genes. (A) rat Cyp2B2; (B) rat Cyp3A7; (C) rat Ugt1A6; (D) human CYP2B6; (E) human CYP3A7; (F) human CYP1A6. **NB**: Red/orange/Green data bars show fold change values significantly (Adjusted p < 0.05) different from control. Blue data bars show non-significant (Adjusted P > 0.05) fold change data values. PB1 = phenobarbital 500 u M; PB2 = phenobarbital 750 u M; PB3 = phenobarbital 1000 u M; PB4 = phenobarbital 2000 u M.

There was a dose- and time-dependent PB-mediated induction of CAR regulated genes CYP2B6, CYP3A7, UGT1A6 and UGT1A8 (Oshida et al 2015, Gardner-Stephen et al 2003, Shelby and Klassen 2006) in human LiMTs and of Cyp2b2, Cyp3a9, Ugt1a6 in rat LiMTs, Fig. 3A–F and Supplementary data Tables 1 and 2, respectively. There was an apparent difference between rat and human LiMTs in the kinetics of onset of CYP3A7 induction which was induced at an earlier time point in rat LIMTs compared to human LiMTs, Fig. 3B and E, respectively. Ugt1a8 RNA was not upregulated by PB in rat LiMTs indicating a species difference with human LiMTs at this level (data not shown). Human isoforms CYP2B6, CYP3A7 and UGT1A6 and rat isoforms Cyp2b2, Cyp3a7 were also induced at the protein level, Supplementary data Tables 1 and 2 respectively. Ugt1a6 was not detected in the proteomics analysis of the rat LiMTs which likely reflects a lower abundance of this isoform at the protein level in rat LiMTs compared to human LiMTs, Supplementary data Table 2.

### 3.3 Pathways analysis

**Fig. 4:**
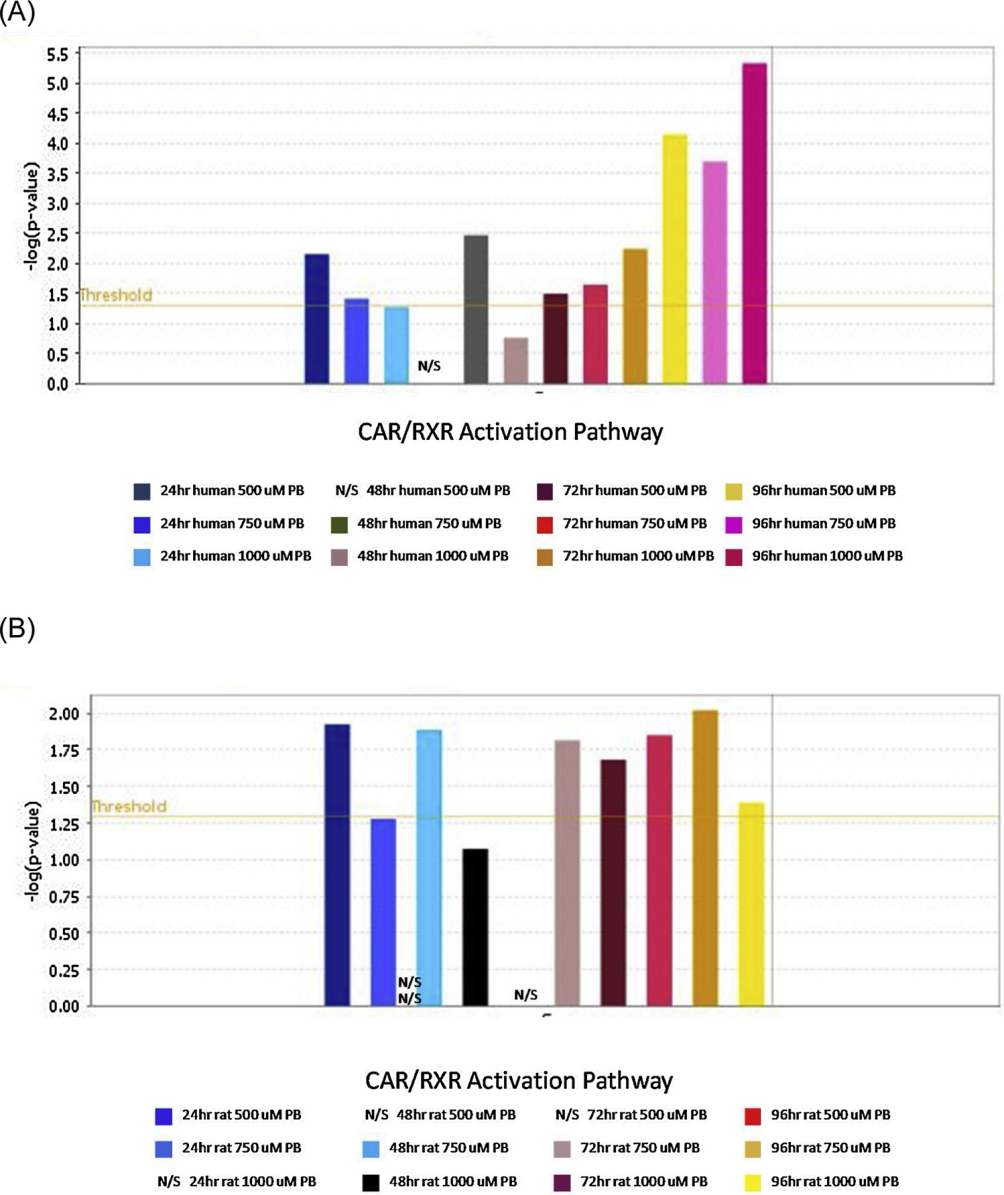
Comparison of over-representation of Ingenuity CAR/RXR ativation pathway genes in the DEG lists derived from a transcriptomic analysis of PB treated human and rat LiMTs. (A) human LiMTs treated with PB; (B) rat LiMTs treated with PB. Histogram columns show false discovery rate (FDR) adjusted p values (-Log p value) for treatments according to PB dose and time. A p value of <0.05 (-log p value >1.3) was considered significant. The orange horizontal line shows this -log p value 1.3 threshold. N/S = not significant.

Integrated transcriptomics and proteomics analysis showed that PB treatment caused differential expression of genes at both the RNA and protein level and the number of differentially expressed genes was for the most part dose-dependant. PCA analysis of the data indicated that both the human and rat data broadly segregates according to treatment, Fig. 1, supplementary data. IPA pathways analysis showed there was a significant (p < 0.05) over-representation of CAR/PXR activation pathways genes in the human and rat LiMT RNA DEG lists at several PB doses at the 24 h, 48 h, 72 h and 96 h time points, Fig. 4A and B, respectively. The CAR/PXR activation pathway genes were mostly induced (2–15 fold) at the RNA level by PB treatment, Supplementary data Tables 1 and 2. Several of the CAR/PXR activation pathway genes were also significantly (P < 0.05) induced at the protein level in both human and rat LiMTs, Supplementary data Tables 1 and 2, respectively. IPA Z score analysis of the RNA DEGs indicated that there was a significant (Z score >2.0) activation of CAR by PB treatments at the 48, 72 and 96 h time points in human LiMTs and at the 48 h and 72 h time points in rat LiMTs, Table 3A and B, respectively. IPA analysis of protein level DEGs also indicated that CAR and PXR were activated, Supplementary data Table 3.

## 4 Discussion

Human and rat LiMTs gave the expected responses at the level of PB-induced hepatocyte proliferation (LI%) in that rat LiMTs showed a significant dose-dependent increase in Li% whereas human LiMTs did not. The data showed fairly high variability reflected in the standard deviation values hence for regulatory studies with new agents it may be necessary to double the number of biological replicates to 8 for this analysis. This data is consistent with previous studies with 2D cultures of primary human and rat hepatocytes [5,6] and indicates that the 3D LiMT model is suitable for assessing human/rat species differences at this level. Our data show that the proliferative response to PB in rat LiMTs is maintained for up to 96 h indicating that rat primary hepatocytes in this 3D model maintain their responsiveness to PB for a longer period than 2D cultures [12]. Previous studies in this LiMT model and also in 2D rat hepatocytes cultured with dexamethazone have shown that the PB induced enzyme induction is maintained for up to 28 days ([11,13], indicating the signalling mechanisms necessary to support enzyme induction responses remains functional when the hepatocytes are grown in 2D or 3D. Our data shows that certain Cyp RNA induction responses of rat LiMTs (e.g Cyp2b2, Cyp3a7) unlike those of human LiMTs (CyP2B6, CYP3A7) appeared to decline at later time points (72 h, 96 h). However there was no indication of a decline in the induction of these isoforms at the protein level as they were both significantly induced at the later time point(s). The reason for the reduced level of induction of certain rat Cyp RNAs at the longer time points by PB is unclear, however our ATP assay data showing similar or slightly increased levels of ATP in PB treated compared to control rat LiMTs suggests that this cannot be attributed to cytotoxicity. The apparent dose-dependent increase in levels of ATP in the rat LIMTs may reflect increased proliferation rates in these tissues. The rat LiMT RNA induction response for Ugt1a6 was maintained across the whole time course.

There was also a species difference in the induction of (UGT1A8) as this UGT isoform was induced by PB in human LiMTs but not in rat LiMTs. Lack of induction of Ugt1a8 by PB in rat LiMTs is consistent with previous in vivo liver RNA induction data for PB with this isoform in rats [14].

There was a significant over-representation of genes in the CAR/RXR activation pathway at all time points in the transcriptomics DEG lists in both rat and human LiMTs and the predicted activation of this CAR pathway was dose- and time-dependant in human LiMTs but not in rat LiMTs (Table 2).

**Table 2:** IPA Z score data for the (A) rat and (B) human CAR receptor (NR1I3). Z scores >2.0 indicate that this transcription factor is significantly activated by the treatments (PB).

**(A)** Rat LIMTs Treatment	Upstream Regulator	Expr Fold Change	Predicted Activation State	Activation z-score	p-value of overlap	Target molecules in dataset
PB 750 u M 48 hr	NR1I3 (CAR)	−1.459	Activated	2.15	0.00188	ALDH1A1,APOA1,CYP2A6,CYP2B6,CYP3A4,CYP3A7,LRG1,TNFRSF1A
PB 750 u M 96 hr	NR1I3 (CAR)	−1.155	Activated	3.374	2.1E-07	ABCB1,ABCC2,AHR,ALDH1A1,APCS,APOA1,CYP2A6,CYP2B6,CYP2C19,CYP2C8
PB 1000 u M 96 hr	NR1I3 (CAR)	−1.127	Activated	2.65	6.24E-05	ABCB1,ABCC2,ALAS1,ALDH1A1,APCS,APOA1,CYP2A6,CYP2B6,CYP2C19,CYP2C8

**(B)** Human LiMTs Treatment	Upstream Regulator	Expr Fold Change	Predicted Activation State	Activation z-score	p-value of overlap	Target molecules in dataset
PB 1000 u M 48 hr	NR1I3 (CAR)	1.712	Activated	2.09	0.0116	ACACA,APOE,CAT,CIDEA,CSF3,GPX1,GSTA5,GSTM5,IL1B,Mt1
PB 750 u M 72 hr	NR1I3 (CAR)	−2.715	Activated	3.203	0.0015	ALAS1,ALDH1A1,CDKN1A,CORO7/CORO7-PAM16,CYP2B6,CYP2C19,CYP2C8,EIF4EBP1,FBRS,GSTM5
PB 1000 u M 72 hr	NR1I3 (CAR)	−2.038	Activated	2.858	0.0147	ALAS1,ALDH1A1,CDKN1A,CYP1A2,Cyp2b13/Cyp2b9,CYP2B6,CYP2C19,CYP2C8,EIF4EBP1,GSTM5

The results of the present study indicate the the 3D LiMT model is fit for the purpose of assessing several key events necessary for establishing a CAR-mediated MOA for rat liver tumours and for assessing the human relevance of this response. Being able to perform these tests in an in vitro model will facilitate a reduction in the use of animals for this purpose.

Integrating the analysis of hepatocyte proliferation and enzyme induction in a single in vitro assay increases the efficiency of a mechanistic investigation, and because the analysis of LIMTs using the microTMA platform uses existing robotic staining and laser dissection technology it is possible to perform tests at an earlier stage in preclinical testing, making this approach suitable for compound screening/lead selection. This could potentially reduce the incidence and costs of adverse liver tumour events attributable to a CAR mode of action in later 2 year bioassay tests. Broadening the pathways analysis process to encompass ‘signatures’ for other nuclear hormone receptors such as peroxisome proliferator activated receptor alpha (PPARA) [15] and aryl hydrocarbon receptor (AHR) [16] would also offer the opportunity to avoid non-human relevant adverse outcomes with other classes of non-genotoxic liver carcinogens. Furthermore since the induction of tumours in other tissues have also been mechanistically linked to activation of nuclear hormone receptors in the liver [[17], [18], [19]], this approach could also reduce the occurrence and associated costs in terms of compound attrition caused by these adverse events.

## Declaration of Competing Interest

The authors declare that they have no known competing financial interests or personal relationships that could have appeared to influence the work reported in this paper. The authors declare the following financial interests/personal relationships which may be considered as potential competing interests.
