# Supplementary material for: Cross-species comparison of CAR-mediated procarcinogenic key events in a 3D liver microtissue model
Source: Toxicol Rep. 2019 Sep 24;6:998–1005. doi: 10.1016/j.toxrep.2019.09.010 (PMC6816142; doi:10.1016/j.toxrep.2019.09.010)
Supplement: Supplementary file 1 [file mmc1.docx]

**Supplementary Data**

| Gene Symbol | 24hr 500 uM PB FC RNA | 24 hr 500 uM PB p-value RNA | 24 hr 750 uM PB FC RNA | 24hr 750 uM PB p-value RNA | 24 hr 1000 uM PB FC RNA | 24hr 1000 uM PB p-value RNA | 48hr 500 uM PB FC RNA | 48hr 500 uM PB p-value RNA | 48 hr 750 uM PB FC RNA | 48hr 750 uM PB p-value RNA | 48 hr 1000 uM PB FC RNA | 48 hr 1000 uM PB p-value RNA | 72 hr 500 uM PB FC RNA | 72hr 500 uM PB p-value RNA | 72 hr 750 uM PB FC RNA | 72 hr 750 uM PB p-value RNA | 72 hr 1000 uM FC RNA | 72 hr 1000 uM PB p-value RNA | 96hr 500 uM PB FC RNA | 96 hr 500 uM PB p-value RNA | 96 hr 750 uM PB FC RNA | 96 hr 750 uM PB p-value RNA | 96 hr 750 uM PB FC protein | 96 hr 750 uM PB p-value protein | 96 hr 1000 uM PB FC RNA | 96 hr 1000 uM PB p-value RNA |
| --- | --- | --- | --- | --- | --- | --- | --- | --- | --- | --- | --- | --- | --- | --- | --- | --- | --- | --- | --- | --- | --- | --- | --- | --- | --- | --- |
| ABCB1 | -1.329 | 0.067 | -1.299 | 0.069 | -1.266 | 0.060 | -1.041 | 0.957 | 1.044 | 0.885 | 1.007 | 0.986 | -1.332 | 0.039 | 1.108 | 0.292 | 1.486 | 0.001 | 2.093 | 0.003 | 3.794 | 0.001 | 2.325 | 0.015 | 3.211 | 0.005 |
| ABCC2 | -1.042 | 0.715 | -1.090 | 0.411 | -1.106 | 0.444 | 1.149 | 0.935 | 1.056 | 0.809 | 1.113 | 0.627 | -1.576 | 0.013 | -1.244 | 0.032 | -1.325 | 0.007 | 1.111 | 0.305 | 1.600 | 0.004 | -1.038 | 0.913 | 1.359 | 0.030 |
| ALDH1A1 | -1.334 | 0.033 | -3.318 | 0.001 | -3.325 | 0.001 | 1.077 | 0.935 | 1.801 | 0.025 | 1.105 | 0.623 | -1.965 | 0.005 | -1.006 | 0.963 | 3.289 | 0.000 | 2.334 | 0.011 | 9.044 | 0.001 | ND | ND | 8.755 | 0.002 |
| CES1 | -15.684 | 0.003 | -39.038 | 0.001 | -47.132 | 0.001 | 1.204 | 0.832 | 3.506 | 0.001 | 2.872 | 0.000 | -10.428 | 0.000 | -1.353 | 0.004 | 3.941 | 0.000 | 2.128 | 0.025 | 8.692 | 0.003 | 1.448 | 0.056 | 8.238 | 0.008 |
| CYP2B6 | -2.095 | 0.005 | -3.417 | 0.002 | -3.547 | 0.004 | 1.065 | 0.935 | 2.187 | 0.019 | 2.173 | 0.000 | -3.962 | 0.001 | 1.656 | 0.001 | 5.266 | 0.000 | 2.914 | 0.001 | 8.205 | 0.000 | 12.205 | 0.036 | 6.667 | 0.004 |
| CYP2C8 | -1.695 | 0.018 | -1.979 | 0.002 | -2.007 | 0.002 | 1.087 | 0.935 | 1.315 | 0.085 | 1.195 | 0.330 | -1.450 | 0.064 | 1.132 | 0.343 | 2.060 | 0.000 | 1.673 | 0.009 | 2.674 | 0.000 | 2.045 | 0.031 | 3.888 | 0.002 |
| CYP2C9 | -1.160 | 0.288 | -1.168 | 0.133 | -1.034 | 0.734 | -1.021 | 0.966 | -1.001 | 0.999 | 1.034 | 0.928 | -2.209 | 0.001 | -1.357 | 0.002 | -1.308 | 0.004 | 1.089 | 0.412 | 1.175 | 0.122 | 1.765 | 0.000 | 1.158 | 0.190 |
| CYP2C18 | -1.748 | 0.033 | -1.948 | 0.004 | -1.959 | 0.005 | -1.083 | 0.935 | 1.041 | 0.858 | -1.001 | 0.998 | -4.375 | 0.001 | -1.338 | 0.005 | -1.161 | 0.068 | 1.102 | 0.621 | 1.858 | 0.011 | ND | ND | 1.877 | 0.045 |
| CYP2C19 | -1.056 | 0.604 | -1.078 | 0.535 | -1.145 | 0.177 | -1.023 | 0.972 | -1.063 | 0.813 | -1.083 | 0.735 | -1.168 | 0.380 | -1.198 | 0.050 | 1.875 | 0.000 | 1.355 | 0.028 | 2.059 | 0.001 | ND | ND | 2.248 | 0.003 |
| GSTA5 | -1.096 | 0.425 | -1.053 | 0.656 | -1.085 | 0.490 | -1.097 | 0.935 | -1.017 | 0.944 | -1.037 | 0.873 | 1.002 | 0.992 | 1.317 | 0.003 | 1.573 | 0.000 | 1.245 | 0.061 | 1.409 | 0.010 | ND | ND | 1.760 | 0.008 |
| MDM2 | 1.081 | 0.500 | -1.052 | 0.615 | 1.356 | 0.526 | -1.104 | 0.935 | -1.178 | 0.340 | -1.113 | 0.593 | -2.005 | 0.002 | -1.726 | 0.000 | -1.752 | 0.000 | -1.065 | 0.500 | 1.432 | 0.015 | ND | ND | 1.451 | 0.032 |
| SULT2A1 | 1.013 | 0.962 | -1.031 | 0.808 | 1.003 | 0.979 | 1.009 | 0.988 | 1.022 | 0.930 | 1.012 | 0.970 | -1.103 | 0.470 | -1.109 | 0.240 | -1.002 | 0.987 | 1.170 | 0.148 | -1.029 | 0.813 | 1.303 | 0.052 | 1.647 | 0.021 |

**Supplementary data Table 1**: Human liver microtissue Integrated transcriptomic and proteomic data showing fold change in response to different doses of phenobarbital (PB) of genes in the CAR/RXR activation pathway. Results are fold change (FC) values (RNA/protein) and false discovery rate (FDR) adjusted p values (q values). ND = Not detected.

| Gene Symbol | 24hr 500 uM PB FC RNA | 24 hr 500 uM PB p-value RNA | 24 hr 750 uM PB FC RNA | 24 hr 750 uM PB p-value RNA | 24hr 1000 uM PB FC RNA | 24hr 1000 uM PB p-value RNA | 48 hr 500 uM PB FC RNA | 48 hr 500 uM PB p-value RNA | 48 hr 750 uM PB FC RNA | 48 hr 750 uM PB p-value RNA | 48 hr 1000 uM PB FC RNA | 48 hr 1000 uM PB p-value RNA | 72 hr 500 uM PB FC RNA | 72 hr 500 uM PB p-value RNA | 72 hr 750 uM PB FC RNA | 72 hr 750 uM PB p-value RNA | 72 hr 750 uM PB FC protein | 72 hr 750 uM PB p-value protein | 72 hr 1000 uM PB FC RNA | 72 hr 1000 uM PB p-value RNA | 96 hr 500 uM PB FC RNA | 96 hr 500 uM PB p-value RNA | 96 hr 750 uM PB FC RNA | 96 hr 750 uM PB p-value RNA | 96 hr 1000 uM PB FC RNA | 96 hr 1000 uM PB p-value RNA |
| --- | --- | --- | --- | --- | --- | --- | --- | --- | --- | --- | --- | --- | --- | --- | --- | --- | --- | --- | --- | --- | --- | --- | --- | --- | --- | --- |
| ABCC2 | -1.224 | 0.174 | 1.319 | 0.049 | -1.014 | 0.941 | -1.390 | 0.499 | -1.157 | 0.666 | 1.278 | 0.353 | 1.037 | 0.930 | -1.487 | 0.036 | ND | ND | -1.700 | 0.017 | 1.121 | 0.276 | 1.361 | 0.010 | 1.546 | 0.001 |
| ABCC4 | -3.404 | 0.006 | -3.377 | 0.004 | -2.590 | 0.050 | -1.078 | 0.965 | -5.910 | 0.074 | 1.406 | 0.685 | 1.067 | 0.981 | 1.324 | 0.831 | ND | ND | 1.355 | 0.813 | -1.351 | 0.003 | -7.545 | 0.000 | -6.363 | 0.000 |
| ALDH1A1 | -20.087 | 0.000 | -5.029 | 0.001 | -4.593 | 0.013 | -1.040 | 0.942 | -5.647 | 0.006 | 3.986 | 0.010 | 2.516 | 0.083 | 6.983 | 0.001 | 1.464 | 0.000 | 14.862 | 0.002 | -2.560 | 0.001 | -3.864 | 0.000 | -3.897 | 0.000 |
| CCND1 | 4.587 | 0.002 | 1.740 | 0.024 | 2.079 | 0.079 | -1.723 | 0.119 | 2.153 | 0.018 | -2.014 | 0.013 | 1.673 | 0.083 | -1.540 | 0.028 | ND | ND | -1.913 | 0.007 | 2.091 | 0.000 | 1.879 | 0.001 | 3.186 | 0.000 |
| Cyp2b2 | -1.423 | 0.037 | 1.277 | 0.107 | -1.347 | 0.151 | 7.845 | 0.044 | -1.621 | 0.138 | 11.884 | 0.007 | 2.982 | 0.065 | 13.930 | 0.001 | 11.048 | 0.000 | 6.910 | 0.005 | -1.670 | 0.001 | -1.651 | 0.004 | -1.730 | 0.001 |
| Cyp2c7 | -4.654 | 0.001 | -2.560 | 0.001 | -2.833 | 0.016 | -1.508 | 0.655 | -9.657 | 0.004 | 3.796 | 0.009 | 2.156 | 0.079 | 10.361 | 0.001 | 1.313 | 0.001 | 16.141 | 0.003 | -3.125 | 0.000 | -6.897 | 0.000 | -5.425 | 0.000 |
| Cyp2c55 | -3.360 | 0.001 | -1.768 | 0.008 | -1.937 | 0.029 | 1.330 | 0.513 | -5.353 | 0.004 | 2.204 | 0.042 | 1.107 | 0.729 | 1.772 | 0.014 | 2.790 | 0.000 | 4.722 | 0.002 | -1.748 | 0.001 | -1.673 | 0.003 | -1.398 | 0.009 |
| GSTA1 | -3.167 | 0.010 | 2.757 | 0.016 | 1.794 | 0.171 | 5.249 | 0.213 | -1.416 | 0.747 | 8.358 | 0.073 | 2.359 | 0.083 | 9.351 | 0.002 | 1.536 | 0.001 | 5.773 | 0.201 | 1.049 | 0.544 | -1.120 | 0.165 | -2.430 | 0.000 |
| CAR (NR1I3) | 2.478 | 0.009 | 1.267 | 0.167 | 1.591 | 0.044 | 1.398 | 0.294 | 2.181 | 0.019 | -3.099 | 0.011 | 1.089 | 0.861 | -2.715 | 0.009 | ND | ND | -2.038 | 0.030 | 2.255 | 0.000 | 2.170 | 0.001 | 2.703 | 0.000 |
| RXRB | 1.898 | 0.024 | 1.084 | 0.575 | 1.091 | 0.738 | 1.141 | 0.802 | 2.071 | 0.042 | -1.217 | 0.406 | 1.642 | 0.172 | -1.933 | 0.012 | ND | ND | -2.046 | 0.014 | 1.303 | 0.005 | 1.496 | 0.003 | 1.467 | 0.002 |

**Supplementary data Table 2**: Rat liver microtissue integrated transcriptomic and proteomic data showing fold change in response to different doses of phenobarbital (PB) of genes in the CAR/RXR activation pathway. Results shown are fold change (FC) values and false discovery rate (FDR) adjusted p values (q values). ND = Not detected.

| **Dose & Species** | **Top Tox Lists^a^** | **p-value** | **Top Canonical Pathways^a^** | **p-value** | **Top Upstream NR Regulators^a^** | **Z-Score of Activation*** | **p-value** |
| --- | --- | --- | --- | --- | --- | --- | --- |
| **PROTEIN** |  |  |  |  |  |  |  |
| **750 uM Rat** | Positive Acute Phase Response Proteins | 3.01E-09 | Acute Phase Response Signaling | 7.81E-08 | AHR | 2.20 | 2.91E-06 |
|  | CAR/RXR Activation | 3.01E-09 | Serotonin Degradation | 1.82E-07 | NR1I2 (PXR) | 2.16 | 2.30E-11 |
|  | Fatty Acid Metabolism | 3.52E-09 | Ethanol Degradation II | 5.27E-07 | N/A | N/A | N/A |
|  | Aryl Hydrocarbon Receptor Signalling | 4.43E-08 | Noradrenaline and Adrenaline Degradation | 7.94E-07 | N/A | N/A | N/A |
|  | LXR/RXR Activation | 4.87E-06 | Coagulation System | 1.02E-06 | N/A | N/A | N/A |
| **750 uM Human** | Xenobiotic Metabolism Signalling | 3.61E-12 | Estrogen Biosynthesis | 2.36E-13 | NR1I3 (CAR) | 2.39 | 2.52E-07 |
|  | Cytochrome P450 Panel - Substrate is a Xenobiotic (Rat) | 1.16E-11 | Nicotine Degradation III | 4.92E-13 | NR1I2 (PXR) | 3.09 | 6.14E-05 |
|  | Fatty Acid Metabolism | 5.56E-11 | Nicotine Degradation II | 1.80E-12 | AHR | 2.22 | 1.54E-11 |
|  | Cytochrome P450 Panel - Substrate is a Xenobiotic (Human) | 1.53E-10 | Melatonin Degradation I | 2.67E-12 | N/A | N/A | N/A |
|  | PXR/RXR Activation | 6.58E-10 | Superpathway of Melatonin Degradation | 6.65E-12 | N/A | N/A | N/A |

**Supplementary data Table 3**: Summary of most significant toxcity pathways, canonical pathways, upstream nuclear receptor regulators and networks associated with the differentially expressed gene lists ( DEGs) caused by PB 750 uM exposure of rat and human liver microtissues at the **protein** level (proteomics data).

^a^ The five most statistically significant toxicity pathways, canonical pathways and upstream nuclear receptor regulators associated with the protein DEGs caused by phenobarbital 750 uM exposure of rat and human liver microtissues. P values are Fisher's exact test p values. The Fisher's exact test algorithm examines the ratio of the number of genes (molecules) related to a particular pathway in a DEG list versus the number genes for that pathway in the IPA database. The algorithm takes into account the total number of genes in the DEG list and the IPA database and calculates the likelihood that an association between the DEG list and the given pathway. * transcription regulator predicted to be activated (z score >2). The IPA z-score algorithm predicts the direction of change for the pathway/regulator. ***Activated*** predictions are only made if the value in the Regulation z‑score column is ≥ 2.  ***Inhibited*** predictions are only made if the value is ≤ ‑2. These predictions are based on the similarity of treatment- induced changes to genes (DEG) in the pathway to changes reported in the literature (IPA knowledgebase) associated with activation/inhibition of the pathway/regulator. N/A = not applicable- (indicates there were less than 5 ligand dependent nuclear receptors showing activation z score >2.0).

Figure 1 Supplementary data: PCA plots of (A) rat and (B) human microarray data.

The rat and human microarray data broadly segregates according to treatment (black ovals).
